# Evaluation of a Simple in-House Test to Presumptively Differentiate *Mycobacterium tuberculosis* Complex from Nontuberculous Mycobacteria by Detection of p-Nitrobenzoic Acid Metabolites 

**DOI:** 10.1371/journal.pone.0080877

**Published:** 2013-11-18

**Authors:** Guirong Wang, Xia Yu, Qian Liang, Suting Chen, Stuart Wilson, Hairong Huang

**Affiliations:** 1 National Clinical Laboratory on Tuberculosis, Beijing Key Laboratory on Drug-resistant Tuberculosis Research, Beijing Chest Hospital, Capital Medical University, Beijing Tuberculosis and Thoracic Tumor Institute, Beijing, China; 2 Microsens Medtech Ltd., London, United Kingdom; Institut de Pharmacologie et de Biologie Structurale, France

## Abstract

The timely differentiation of *Mycobacterium tuberculosis* complex (MTC) and non-tubercular mycobacterium (NTM) species is urgently needed in patient care since the routine laboratory method is time consuming and cumbersome. An easy and cheap method which can successfully distinguish MTC from NTM was established and evaluated. 38 mycobacterial type and reference strains and 65 clinical isolates representing 10 species of mycobacterium were included in this study. Metabolites of p-nitrobenzoic acid (PNB) reduction were identified using liquid chromatography and tandem mass spectrometry (LC/MS/MS). A spectrophotometric method was developed to detect these metabolites, which was evaluated on a number of MTC and NTM species. All of the tested NTM species and strains reduced PNB to p-aminobenzoic acid (PABA), while none of the MTC strains showed a similar activity. Spectrophotometric detection of PABA had 100% sensitivity and specificity for MTC and NTM differentiation among the type strains and the clinical isolates tested. PABA was identified as one of the metabolites of PNB reduction. All the tested NTM species metabolized PNB to PABA whereas the MTC members lacked this activity. A simple, specific and cost-effective method based on PABA production was established in order to discriminate MTC from NTM from cultured organisms.

## Introduction

Tuberculosis (TB) is one of the major causes of morbidity and mortality worldwide. Tuberculosis in humans is primarily caused by bacterial special of the *Mycobacterium tuberculosis* complex (MTC), however, infections due to a range of nontuberculous mycobacteria (NTM) have been reported to be increasing [1]. The majority of patients with MTC infection can be successfully cured with primary anti-TB drugs, on the contrary many NTM are resistant to the commonly used anti-TB drugs [2]. Thus, rapid and precise differentiation of MTC from NTM infections is essential for appropriate treatment. 

Conventional tests to differentiate MTC from NTM are mostly based on using different inhibitors such as hydroxylamine hydrochloride (HA), 8-azaguanine [3], sodium salicylate, p-nitro-α acetylamino β hydroxypropiophenone (NAP) [4,5], nitroxoline [6], and propylene glycol [7]. Many of these methods are technically demanding, time-consuming and require specialized reagents. In addition, ambiguous results have been reported when using these tests [8]. Other methods such as molecular probes and high performance liquid chromatography (HPLC) have been proposed for mycobacterial species differentiation but these methods are technically laborious and expensive which prevents them from being applied in laboratories with limited resources [9]. 

Para-nitrobenzoic acid (PNB) [10] is a commonly used selective inhibitor of MTC and at 500μg/ml in media inhibits the growth of MTC strains, whereas NTM strains are resistant. However, the reporting time of this inhibition test by BACTEC MGIT960 system ranged from 4-11 days (median 5.9 days) for MTC strains and 2-10 days (median 4.5 days) for NTM strains [8]. Analysis by solid LJ culture can take even longer, with more than 20 days reported [9]. Although this inhibition test has been used for more than 50 years, its exact working principle remains unclear. The objective of the present study was to investigate the metabolism of PNB in order to develop a reliable, easy and inexpensive test for the differentiation of MTC and NTM.

## Materials and Methods

### Microorganisms

In total 38 type and reference strains of the genus mycobacterium, including 5 MTC members and 33 NTM species, 65 clinical isolates representing 32 *M .tuberculosis* strains, 5 *M. abscessus* strains, 5 *M. avium* strains, 5 *M. fortuitum* strains, 5 *M. gordonae* strains, 5 *M. intracellulare* strains, 5 *M. kansassi* strains, 1 *M. malmoense* strains, 1 *M. parascrofulaceum* strains, 1 *M. scrofulaceum* strains were investigated in this study. The clinical mycobacterial isolates were identified to species level by sequences alignment of *16S rDNA*, *16-23S rRNA* gene internal transcribed spacer (ITS) and *rpoB* gene. 

### PNB reduction test

Recently grown colonies were scraped from the surface of the LJ media, weighed and emulsified by vortexing in flasks containing glass beads. The bacilli concentration was adjusted to 1.6mg/ml with water. 1ml of the suspension was transferred to a new tube and 5μl 5% PNB (Sigma-Aldrich, St Louis, MO, USA) was added and incubated at 37°C overnight. Each suspension was then filtered through a 0.2-μm-pore-size Millipore syringe filter before applying to HPLC or to chromatography/tandem mass spectrometry (LC/MS/MS). Reactions without bacilli or PNB substrate were used as controls.

### Identification of the metabolites of PNB by HPLC

Five microliters of the filtered solution from the overnight PNB incubation was analyzed by HPLC (L-6200 Intelligent pump, L-4200 UV-vis detector, 655A-40 autosampler, 655A-52 column oven, and D-2000 Chromato-Integrator [Hitachi, Japan] and a Chrom C18 column [3.9 ×150 mm, 5μm;Waters]). The fluid phase consisted of methanol: phosphate buffer (pH 3.52) (1:1). Chromatography was performed at room temperature at a flow rate of 1.0 ml/min with a UV detector at 275 nm. 3μg/ml PNB solution in methanol and 3μg/ml p-aminobenzoic acid (PABA, Sigma-Aldrich, St Louis, MO, USA) solution in water were used as standards. To investigate the ionization of the compound and for optimization of the compound-specific parameters, mixture of PNB and PABA solution (1:1) was analyzed by HPLC. 

### Identification of the metabolites of PNB by LC/MS/MS

Two microliters of the filtered solution from overnight PNB incubation was analyzed by HPLC (Agilent 1200,Palo Alto, CA, USA) using Agilent ZORBAX SB-Aq column (2.1mm×100mm,3.5μm) guarded by a ZORBAX SB-Aq column (2.1mm×12.5mm,5μm). The mobile phase consisted of 0.1% formic acid:acetonitrile (40:60, v/v) and the column was kept at 30°C. 

The assay was then performed on an Agilent G6410B tandem quadruple mass spectrometer by positive ion electrospray ionization in the positive multiple reaction monitoring (MRM) mode. The transition of m/z was 138→77.1 for PABA. The analysis completed within 5 min. 

A PABA stock solution was serially diluted in water to prepare standard solutions ranging from 25-1600ng/ml; five different concentrations were used for developing a calibration curve. The concentration of PABA in each tested sample was calculated on the basis of calibration curve.

### PNB metabolite detection by spectrophotometry

All of 38 type strains and 65 clinical strains were tested. The PNB reduction tests were performed as described above but using bacilli at 20mg/ml. After the incubation, the reactions were processed as described [11]. Briefly, 450μl of 15% trichloroacetic acid, 1.3ml of H_2_0 and 250μl of 0.1% sodium nitrite were added to one ml of supernatant and incubated 15 minute on ice. 250μl of 0.5% ammonium sulfamate was then added and incubated on ice for 3 minutes. Finally, 1.5ml of 0.1% N-(1-Naphthyl) ethylendiamine dihydrochloride (NEDD) was added. The diazonium salt formed from the PABA couples with NEDD in the acidic solution and also produced a red-purple azo dye which could be measured by absorbance at OD540nm. Each sample was tested in triplicate, and the data were presented as average. 5 different concentrations of PABA were used to develop the calibration curve and the amount of PABA produced was calculated according to the calibration curve. 

Additionally, we also investigated the minimal bacilli concentration which is needed for the PNB metabolite detection by spectrophotometry. Different bacilli concentrations (20mg/ml, 10mg/ml, 5mg/ml, and 2.5mg/ml) of *M. fortuitum* and *M. avium* type strains were analyzed. The minimal incubation time was also investigated by incubating *M. avium* and *M. smegmatis* type strains at 20mg/ml for different time points.

### Statistical analysis

The Student t-test was conducted to assess statistical significance between groups of strains. P<0.05 was considered to be statistically significant. 

## Results

### Metabolites of PNB reduction analyzed by HPLC

**Figure 1:**
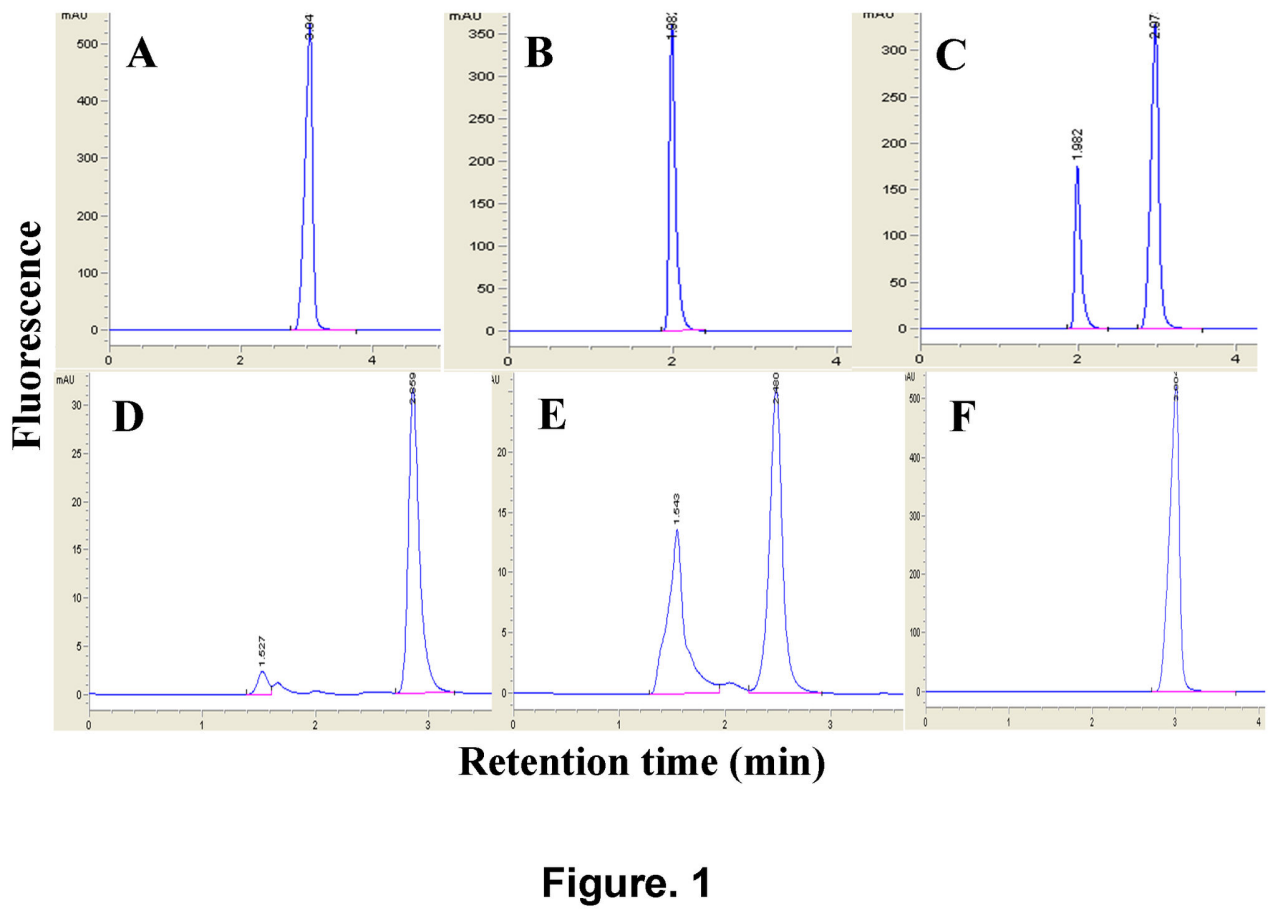
HPLC outcomes. (A) PNB standard, (B) PABA standard, (C) PNB and PABA mixed standards (D) representative sample of *M. smegmatis*, (E) representative sample of *M. intracellulare*, and (F) representative sample of *M. tuberculosis* H37Rv.

For HPLC, retention time for the PNB standard was 3.04min and for PABA it was 1.98min. The mixture of PNB and PABA solution (1:1) gave a retention time of 2.97min and 1.98min respectively (Figure 1). All the PNB reduction tests for the 32 NTM type strains produced double peaks with retention times of 1.5min and 2.6min. For non-bacilli controls or reactions involved 5 MTC members, only one peak at 3min was observed, while the non-substrate control did not produce any peak (Figure 1). These results suggested that PABA might be one of the metabolite for PNB reduction.

### Concentration of PABA of standard strains analyzed by LC/MS/MS

**Figure 2:**
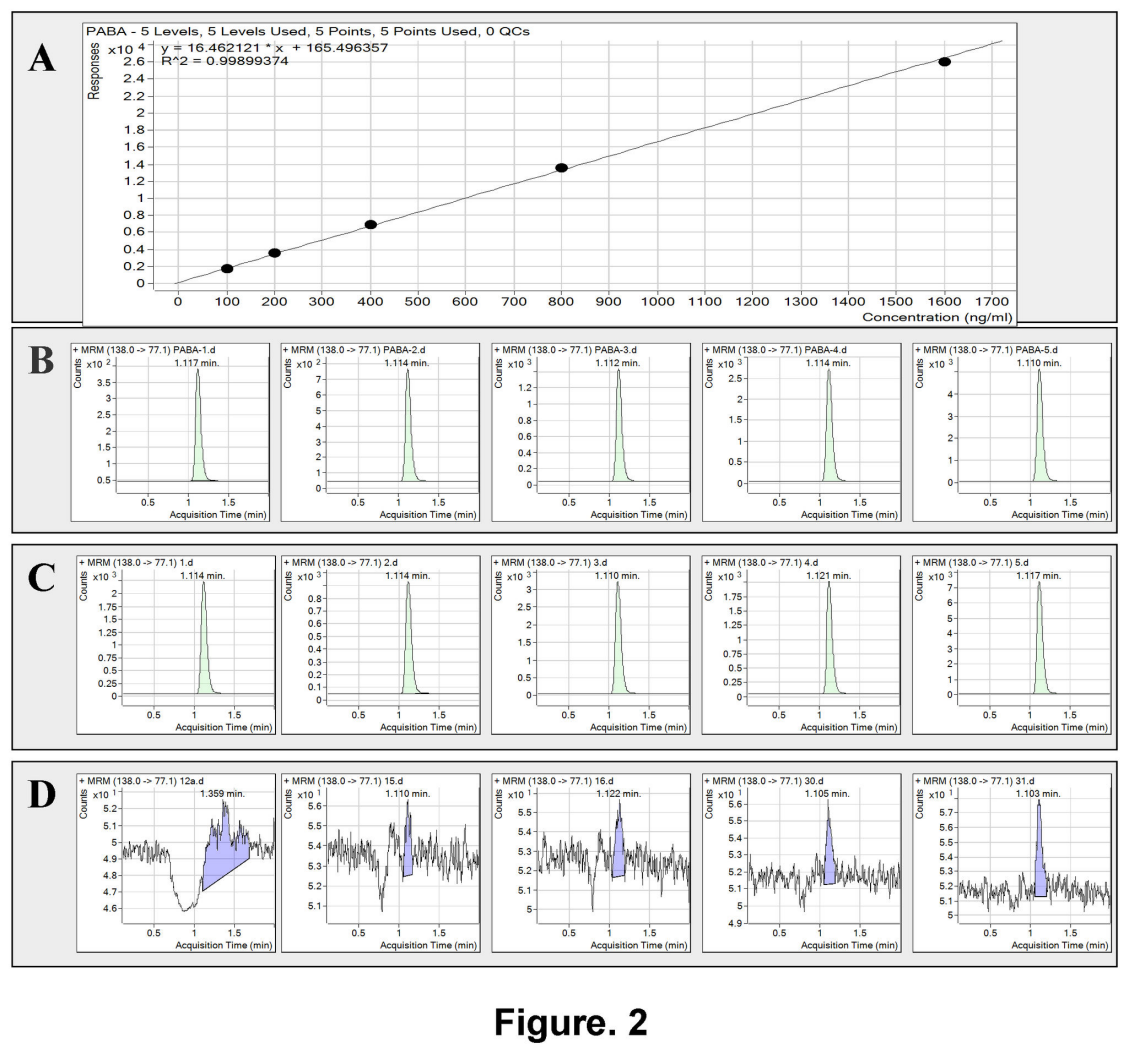
Calibration curves of PABA and representative LC-MS/MS outcomes for some analyses: (A) calibration curves of PABA. Equations of linear regression curve fitting and coefficients of determination are shown above. (B) LC-MS/MS outcomes of 5 concentration of PABA (from left to right representing: 100ng/ml, 200ng/ml, 400ng/ml, 800ng/ml, 1600ng/ml,). (C) LC-MS/MS outcomes of 5 NTM species (from left to right representing: *M. abscessus*, *M. avium*, *M. fortuitum*, *M. gordonae*, *M. intracellulare*). (D) LC-MS/MS outcomes of 5 MTC members (from left to right representing: *M. tuberculosis* H37Rv, BCG, *M.bovis*, *M. africanum*, *M. microti*).

All of the 32 NTM type strains investigated in our assay presented a peak at similar retention time to that of PABA control. Neither the 5 MTC members nor the no-bacilli or no-PNB substrate controls produced notable peak at that time point. Our outcomes strongly suggested that the metabolite responsible for PNB reduction by NTM was PABA (Figure 2). The concentrations of PABA in the reactions detected by LC/MS/MS were calculated and are shown in Table 1. 

**Table 1:** Concentration of PABA in the reactions of the 38 type strains analyzed by LC/MS/MS.

ATCC number	Strain name	PABA concentration (μg/ml)
19274	BCG	0.00
27294	*M. tuberculosis* H37Rv	0.00
19210	*M. bovis*	0.00
35711	*M. africanum*	0.00
19422	*M. microti*	0.00
27023	*M. obuense*	0.11
13950	*M. intracellulare*	0.12
27278	*M. chubuense*	0.12
35752	*M. chelonae*	0.13
23366	*M .aurum*	0.13
19420	*M. smegmatis*	0.17
25291	*M. avium*	0.20
35796	*M. senegalense*	0.21
19250	*M. xenopi*	0.22
33027	*M. sphagni*	0.22
12478	*M. kansassi*	0.24
14474	*M. flavescens*	0.25
43909	*M. gilvum*	0.25
19527	*M. thermoresistibile*	0.29
19340	*M. diernhoferi*	0.29
27282	*M. tokaiense*	0.33
33776	*M. porcinum*	0.34
6841	*M. fortuitum*	0.38
43910	*M. duvalii*	0.40
19619	*M. terrae*	0.40
27024	*M. rhodesiae*	0.41
14472	*M. chelonae*	0.42
19977	*M. abscessus*	0.44
15754	*M. gastri*	0.49
15483	*M. vaccae*	0.49
19688	*M. parafortuitum*	0.51
25276	*M. asiaticum*	0.57
27280	*M. aichiense*	0.59
25795	*M. neoaurum*	0.74
33464	*M. austroafricanum*	0.78
14470	*M. gordonae*	0.95
25799	*M. szulgai*	1.11
927	*M. marinum*	1.49

### Concentration of PABA in the PNB reduction test of the clinical isolates analyzed by spectrophotometry

 Among all test strains, including type strains and clinical strains, the A540 of MTC strains was lower than 0.04; while those of NTM strains was higher than 0.1 (P<0.05, P=2.61×10^-7^). When the cut-off value of A540 was set up at 0.07, the spectrophotometric detection of PABA had 100% sensitivity and specificity for MTC and NTM differentiation among the type strains and the clinical isolates tested.

**Figure 3:**
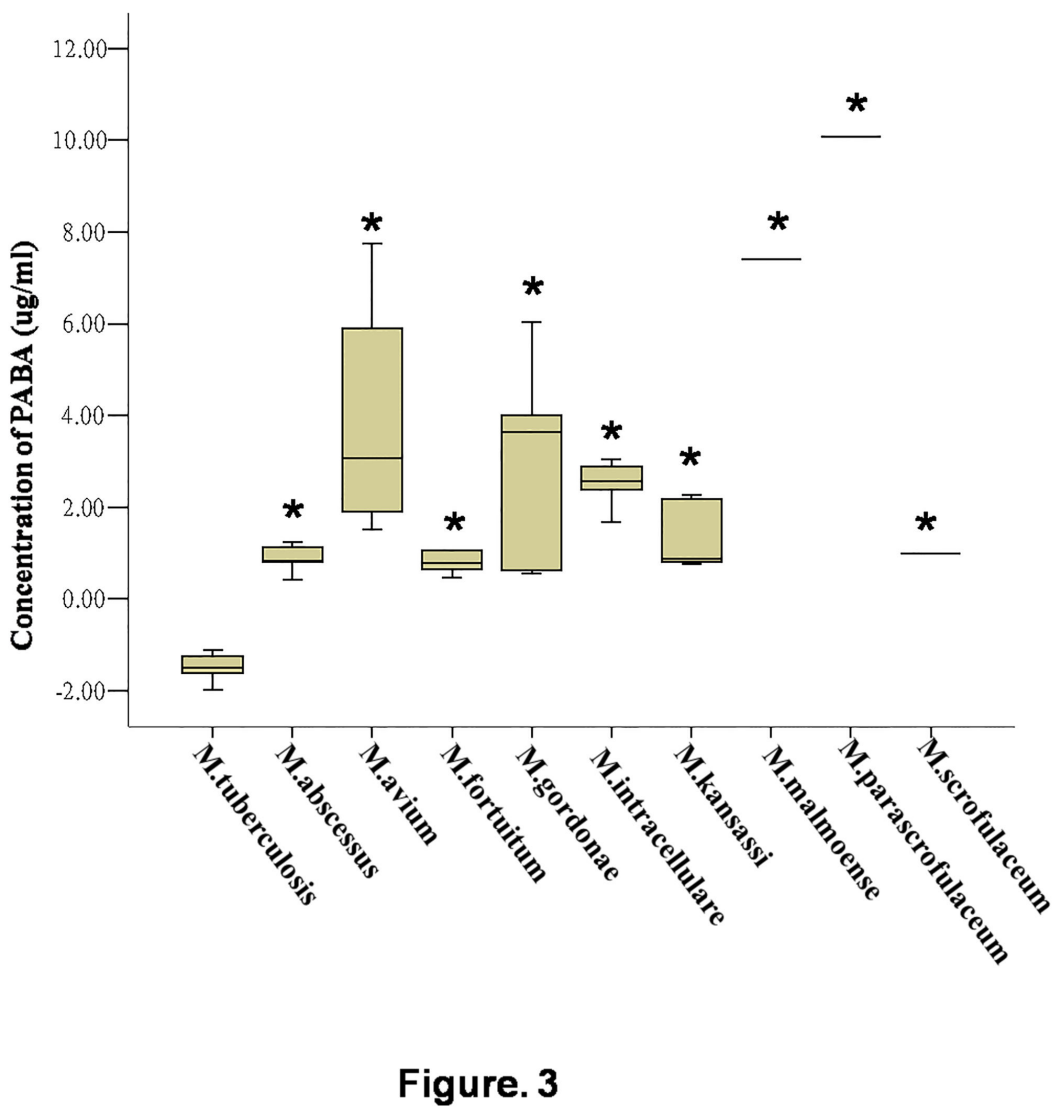
Scattergraph of PABA production of clinical strains analyzed by spectrophotometer. The concentrations of PABA (μg/ml) production are shown on y axis. Boxes represent 25th, 50th and 75th percentiles of the data. The length of the box is the interquartile range. * represents statistically significant (P<0.05) when compared with *M. tuberculosis*.

After calculating the PABA concentration according to the calibration curve, the amount of PABA produced by 32 *M .tuberculosis* clinical isolates was found to be negligible, while all the NTM strains were able to produce some PABA. Concentrations of PABA produced by the clinical isolates analyzed by spectrophotometry are presented in Figure 3.

**Figure 4:**
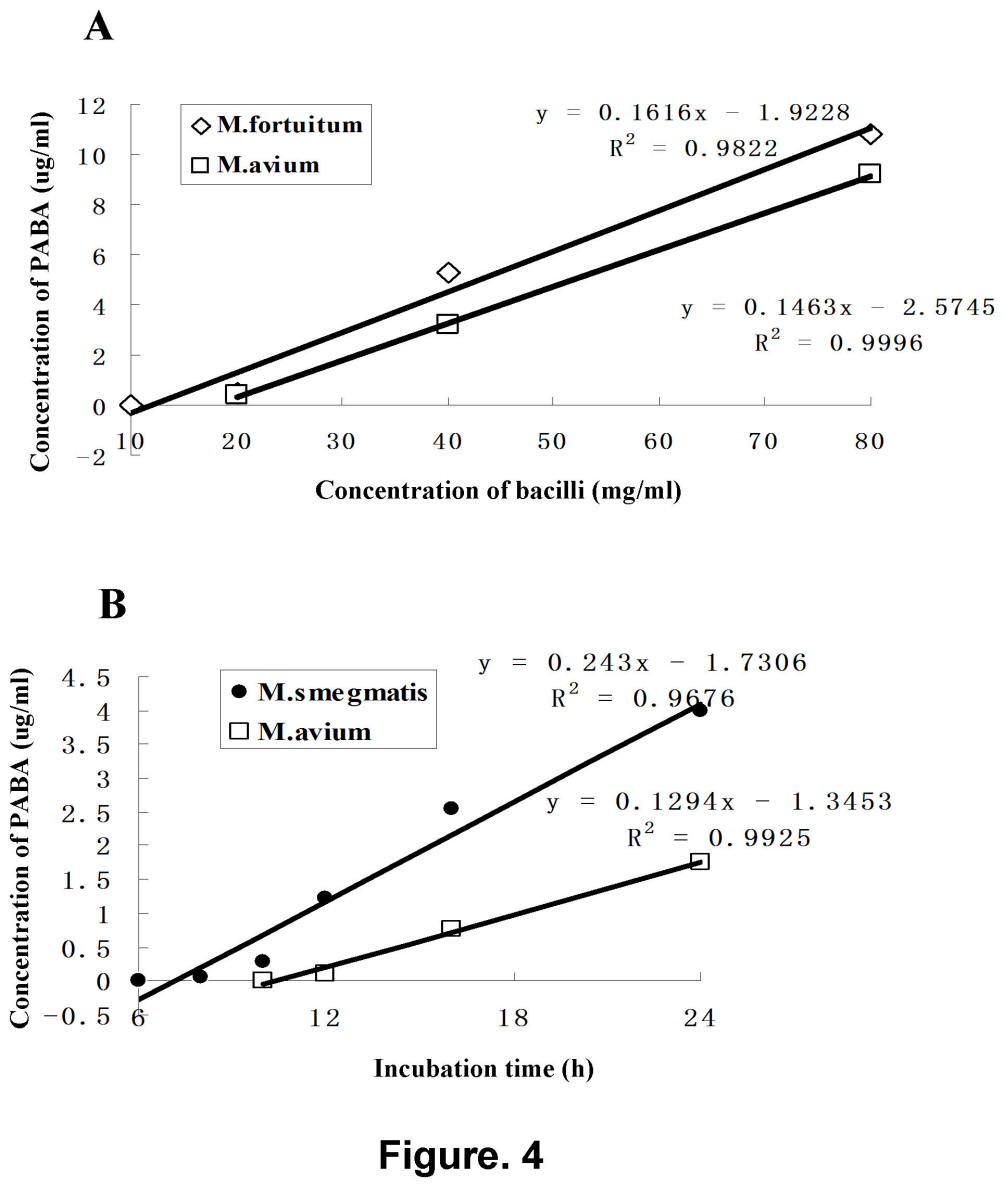
Correlation between concentration of bacilli and PABA production (A), and correlation between incubation time and PABA production (B). Linear regression equations and coefficients of determination are demonstrated.

PABA production increases with longer incubating time and higher bacilli concentration (Figure 4). The minimal bacilli concentration required for the PNB reduction test for both *M. fortuitum* strain and *M. avium* strains was 20mg/ml when detected by spectrophotometry after overnight incubation. The minimal incubation time for slow-growing *M. avium* was 12h and for rapid-growing *M. smegmatis* was 8h when using 20mg/ml bacilli.

## Discussion

MTC and NTM are clinically similar in terms of producing diseases and diagnostic delay can affect the treatment. It is important to identify the infecting mycobacterium to the species level, but a preliminary differentiation between MTC and NTM is more practical in clinical laboratories. The commonly used differentiation methods between MTC and NTM include conventional biochemical tests, MPB64 antigen detection and microscopic observation of cord formation after Ziehl Neelsen staining [12-14]. While Chihota et al [15] reported the cost of organism identification per positive culture on BACTEC as US$35.94 using standard biochemical tests, US$15.49 for anti-MPB64 assay and US$2.28 for cording assay. Although the cording assay is cheap and fast, the reliability of the assay is heavily dependent on the experience of the microscopists, and the frequent dubious outcomes limit this assay to be only used as a preliminary screening test [16]. The anti-MpB64 assay is simple and rapid, but routine performance proved it cannot differentiate certain species correctly and some *M. tuberculosis* stains harboring mpb64 gene mutation can yield false negative results, which is another concern [17,18]. In this study, we established a simple and cost-effective in-house differentiation test for *Mycobacterium tuberculosis* complex and non-tuberculous mycobacteria by detecting PABA in a simple activity assay. This test is intended for cultured microorganisms just as the other differentiation methods aforementioned, and usually need days or even weeks before enough bacilli could be harvested [19]. The minimal bacilli needed for the PABA assay is about 1mg per reaction, which is about one full loop of bacilli. Considering the quantity of bacilli needed for the PABA detection, it may need a few more days on culture to get enough bacilli than cord assay and anti-MPB64 test dose, but requires less time than biochemical tests dose. We tested several BACTEC positive samples with PABA detection assay, and found the samples had sufficient bacilli for the assay (data not shown). After obtaining cultured organisms, the presumptive identification of MTC or NTM could be done within 8h to overnight by our assay. When the cost of culture is excluded, the cost of this identification test alone was much less than US$1 per test, and the only machine required is a spectrophotometer which is often standard equipment in clinical laboratories. Among the tested type strains and clinical strains, the method showed 100% sensitivity and specificity. However, since we did not test the clinical samples blindly, we could not calculate the positive and negative predictive value of the test this time. Since MTC is the most common mycobacterial infection in developing countries, this method could be used preliminarily to differentiate MTC from NTM. After this differentiation, speciation could be achieved by some other sophisticated and high discriminatory methods such as DNA sequencing.

In our study, we detected PABA in all the reactions of NTM strains, including 33 NTM type strains and 33 NTM clinical strains representing 9 species, but none of the MTC strains, including 5 classical MTC species (*M. tuberculosis, M. africanum, M. bovis, M. bovis BCG and M. microti*) and 32 MTC clinical strains. We did not include any of the newly identified MTC members (such as *M. caprae, M. pinnipedii and M. canettii*) due to unavailability of sufficient strains. As these strains do have a little clinical significance in relation to humans, and we wish to analyze them in the near future. Furthermore, this test could not identify coinfections due to MTC and NTM, a phenomenon which is relatively less common and a complicated clinical condition. 

In 1964, Tsukamura [7] reported that PNB could be reduced to PABA by *M. avium*. However, no other reports indicate that *M. avium* or other NTM strains can produce PABA, and the mechanism of PNB tolerance by NTM is still barely known. In order to investigate the mechanism, we detected the metabolites of PNB by mycobacterium species and strains using HPLC and LC/MS/MS. We found that none of the five MTC species or strains could reduce PNB to PABA whereas all NTM strains that we tested have the ability to reduce PNB to PABA, which probably explains the basis of the PNB inhibition test. It is known that PNB is toxic [20], whereas PABA is non-toxic and is an intermediate product of folic acid synthesis, which is an important reaction for bacterial survival [21].

In conclusion, we identified that NTM but not MTC species and strains could reduce PNB to PABA, which might be the mechanism behind the PNB inhibition test for differentiation of MTC from NTM. We also developed a simple, reliable and cost-effective in-house differentiation test for MTC and NTM, and this test can be easily used in clinical laboratories in combination with solid culture to help in appropriate management of tuberculosis.
